# MicroRNA-212 suppresses tumor growth of human hepatocellular carcinoma by targeting FOXA1

**DOI:** 10.18632/oncotarget.3916

**Published:** 2015-04-23

**Authors:** Changwei Dou, Yufeng Wang, Chao Li, Zhikui Liu, Yuli Jia, Qing Li, Wei Yang, Yingmin Yao, Qingguang Liu, Kangsheng Tu

**Affiliations:** ^1^ Department of Hepatobiliary Surgery, The First Affiliated Hospital of Xi'an Jiaotong University, Xi'an, China

**Keywords:** MiR-212, FOXA1, hepatocellular carcinoma, proliferation, apoptosis

## Abstract

MicroRNA-212 (miR-212) has been reported to play oncogenic or tumor suppressive role in different human malignancies. Here, we demonstrated that the mean level of miR-212 in hepatocellular carcinoma (HCC) tissues was significantly lower than that in matched tumor-adjacent tissues. Similarly, the expression of miR-212 was obviously reduced in HCC cell lines as compared with a nontransformed hepatic cell line. Ectopic expression of miR-212 inhibited cell viability and proliferation, and induced apoptosis in HepG2 cells. In contrast, down-regulation of miR-212 increased cell viability and proliferation, and suppressed apoptosis in Bel-7402 cells. *In vivo* studies showed that miR-212 inhibited tumor growth of HCC via suppressing proliferation and inducing apoptosis. Furthermore, we confirmed that Forkhead box protein A1 (FOXA1) was a direct target of miR-212, and it abrogated the function of miR-212 in HCC. Finally, we disclosed that the aberrant expression of miR-212 and FOXA1 was evidently correlated with poor prognostic features of HCC. MiR-212, FOXA1 and their combination were valuable prognostic markers for predicting survival of HCC patients. In conclusion, miR-212 may serve as a prognostic indicator for HCC patients and exerts tumor suppressive role, at least in part, by inhibiting FOXA1.

## INTRODUCTION

MicroRNAs (miRNAs) are a class of evolutionarily conserved noncoding RNAs that act as post-transcriptional regulators of gene expression through interacting with the 3′-untranslated region (3′-UTR) of target messenger RNAs (mRNAs). They participate in various biological processes [1] including proliferation, apoptosis, cell cycle, and stem cell renewal. Abundant investigations have confirmed that deregulation of miRNAs contributes to the development and progression of human malignancies [2] including hepatocellular carcinoma (HCC). Furthermore, miRNAs are closely associated with the diagnosis, clinical features and survival of patients [3]. Increasing studies have confirmed that miRNAs play critical roles in the proliferation [4], apoptosis [5], angiogenesis [6, 7] and metastasis [8] of HCC. Therefore, miRNAs have been proposed as promising prognostic markers and attractive therapeutic targets for HCC patients [9, 10].

MiR-212, which is located at chromosome 17p13.3 [11], has been shown to be deregulated in various human cancers. MiR-212 serves as a tumor suppressor in non-small cell lung cancer (NSCLC) [12] and gastric carcinoma [13]. However, other studies suggest that miR-212 exhibits oncogenic properties in colorectal cancer [14], prostate cancer [15] and pancreatic cancer [16]. Therefore, the biological functions of miR-212 are cancer-type specific, partly resulted from the different cellular contexts of various tumors. Different from miR-122 [17-19], which has been actively studied in HCC, miR-212 has not been investigated in detail in HCC.

Forkhead box protein A1 (FOXA1) is a member of FOXA gene family, and is an important regulator of proliferation, apoptosis and cell cycle. It plays oncogenic roles and has been considered as a predictor of poor survival in anaplastic thyroid cancer [20], prostate cancer [21] and triple-negative breast cancer [22]. FOXA1 promotes hepatocarcinogenesis in male mice and is responsible for the sexual dimorphism of HCC [23]. FOXA1 has been reported to promote the expression of Yes-associated protein (YAP), which is a terminal effecter of Hippo/YAP signaling and contributes to cell proliferation of liver cancer [24]. Therefore, FOXA1 is an important contributor of HCC development. But the clinical significance and regulatory mechanism of FOXA1 expression in HCC are poorly understood.

In this study, we found that miR-212 was down-regulated in the majority of HCC tissues. *In vitro* and *in vivo* studies demonstrated that miR-212 suppressed cell viability and proliferation, and induced apoptosis in HCC cells. Moreover, we revealed that miR-212 exerted its biological function, at least in part, by inhibiting FOXA1 expression. Notably, miR-212, FOXA1 and their combination are valuable predictors for the prognosis of HCC patients. Our results elucidate the underlying mechanism by which miR-212 inhibits HCC, and propose miR-212 as a potential therapeutic target for HCC.

## RESULTS

### The expression of miR-212 is down-regulated in HCC tissues and cell lines

**Figure 1:**
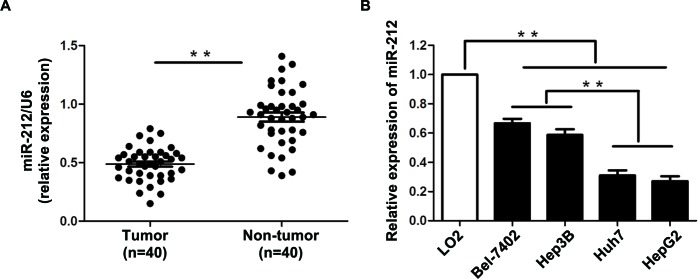
The expression level of miR-212 in HCC tissues and cell lines **A.** The expression of miR-212 in HCC tissues was significantly lower than that in matched tumor-adjacent tissues. ** *P* < 0.01 by *t* test. **B.** Comparing differences in the expression levels of miR-212 between HCC cell lines with different proliferative potentials and the nontranformed hepatic cell line LO2. *n* = three independent experiments, ** *P* < 0.01 by ANOVA.

To determine the expression status of miR-212 in HCC, we initially compared the expression of miR-212 in 40 pairs of HCC tissues and adjacent non-tumor tissues. The expression of miR-212 in HCC tissues was significantly lower than that in matched tumor-adjacent tissues (*P* < 0.01, Figure 1A). Next, we evaluated the relative expression of miR-212 in a nontransformed hepatic cell line (LO2) and a panel of human HCC cell lines (Bel-7402, Hep3B, Huh7 and HepG2). Reduced expression of miR-212 was observed in all four HCC cell lines as compared with LO2 (*P* < 0.01, Figure 1B). Notably, the expression of miR-212 in Bel-7402 and Hep3B cells was higher than that in Huh7 and HepG2 cells (*P* < 0.01, Figure 1B). These data indicate that reduced level of miR-212 may be involved in the development of HCC.

### MiR-212 inhibits HCC cell proliferation and induces apoptosis *in vitro* and *in vivo*

**Figure 2:**
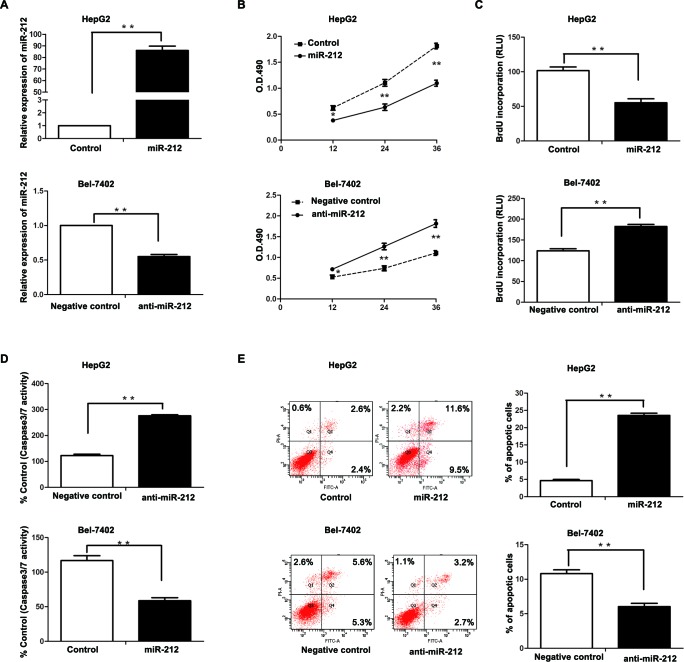
The effect of miR-212 on HCC cell proliferation and apoptosis **A.** MiR-212 mimics significantly increased the expression of miR-212, while miR-212 inhibitors effectively decreased miR-212 expression, as determined by qRT-PCR. ** *P* < 0.01 by *t* test. **B-E.** Altering expression of miR-212 significantly affected the cell viability, proliferation, caspase-3/7 activity, and the percentage of apoptotic cells in HepG2 and Bel-7402 cells. *n* = three repeats with similar results, * *P* < 0.05, ** *P* < 0.01 by *t* test (BrdU incorporation, caspase-3/7 activity and flow cytometry assays) and ANOVA (MTT assay).

To further explore the biological role of miR-212 in HCC, a miR-212 expression vector and a miR-212 inhibitor (anti-miR-212) was transfected into HepG2 and Bel-7402 cells, respectively. As measured by qRT-PCR, miR-212 expression vector significantly increased the level of miR-212 in HepG2 cells (*P* < 0.01, Figure 2A), while the anti-miR-212 vector significantly reduced the expression of miR-212 in Bel-7402 cells (*P* < 0.01, Figure 2B). MTT and BrdU assays demonstrated that forced expression of miR-212 in HepG2 cells (HepG2-miR-212) resulted in significant decrease of cell viability and proliferation (*P* < 0.01, respectively, Figure 2B and 2C), while down-regulation of miR-212 in Bel-7402 cells (Bel-7402-anti-miR-212) showed remarkable increase of cell viability and proliferation as compared with control cells (*P* < 0.01, respectively, Figure 2B and 2C). Moreover, as determined by flow cytometry and caspase 3/7 activity assays, miR-212 overexpression increased the percentage of apoptotic HepG2 cells (*P* < 0.01, for both assays, Figure 2D and 2E) and its down-regulation inhibited apoptosis in Bel-7402 cells (*P* < 0.01, for both assays, Figure 2D and 2E).

**Figure 3:**
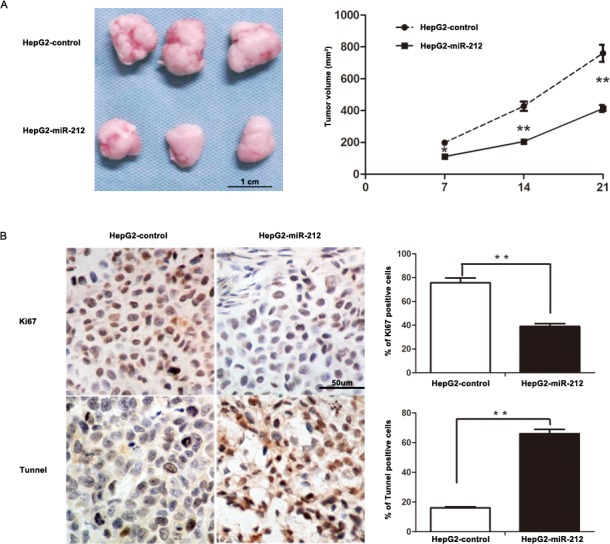
MiR-212 slows down tumor growth in mice **A.** Tumor growth curve revealed that miR-212 overexpression significantly inhibited tumor growth *in vivo*. *n* = six, ** *P* < 0.01 by ANOVA. **B.** Up-regulation of miR-212 inhibited proliferation and induced apoptosis *in vivo*. The photomicrographs for Ki-67 staining and TUNEL assays were shown. The percentage of Ki-67 positive cells in tumors arising from HepG2-miR-212 group was significantly lower than that arising from HepG2-miR-control group. The percentage of apoptotic cells in the HepG2-miR-212 group was significantly higher than that in the HepG2-miR-control group. *n* = six, * *P* < 0.05, ** *P* < 0.01 by *t* test.

To further confirm these results *in vitro*, HepG2 cells that were transfected with miR-212 or control vector were implanted subcutaneously into nude mice. Tumor growth curves, generated over 21 days, revealed that up-regulation of miR-212 significantly slowed down tumor growth in mice (*P* < 0.01, Figure 3A). Importantly, the isolated tumor tissues were subjected to immunohistochemistry for Ki67 and TUNEL assays. Our data confirmed that forced expression of miR-212 inhibited cell proliferation and induced apoptosis *in vivo* (*P* < 0.01, respectively, Figure 3B). These results indicate that miR-212 adversely affects tumor growth of HCC by inhibiting cell viability and proliferation, and inducing apoptosis.

### FOXA1 is a direct downstream target of miR-212

**Figure 4:**
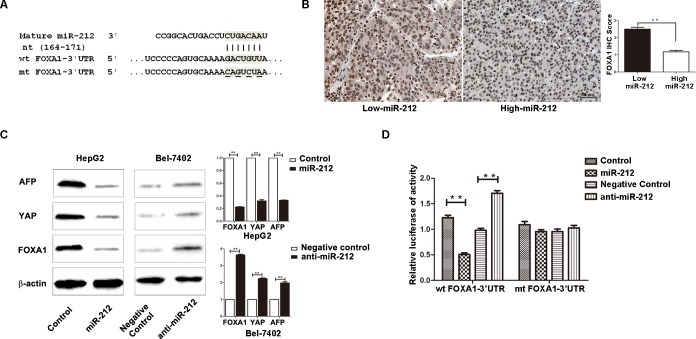
FOXA1 is a direct downstream target of miR-212 in HCC **A.** MiR-212 and its putative binding sequence in the 3′-UTR of FOXA1. The mutant miR-212 binding sites were generated in the complementary site for the seed region of miR-212 (wt, wild type; mt, mutant type). **B.** Inverse relationship between the expression of miR-212 and FOXA1 was found in HCC specimens. *n* = 40, ** *P* < 0.01 by *t* test. **C.** Up-regulation of miR-212 in HepG2 cells significantly inhibited the expression of FOXA1, AFP and YAP. Down-regulation of miR-212 in Bel-7402 cells significantly increased the expression of FOXA1, AFP and YAP. *n* = three repeats with similar results, ** *P* < 0.01 by *t* test. **D.** miR-212 overexpression significantly suppressed the luciferase activity that carried wild type but not mutant type 3′-UTR of FOXA1. Downregulating miR-212 increased the luciferase activity that carried wild type but not mutant type 3′-UTR of FOXA1. *n* = three repeats with similar results, ** *P* < 0.01 by *t* test.

To find out the molecular mechanism responsible for the biological functions of miR-212 in HCC cells, we searched for candidate target genes of miR-212 by using public databases including TargetScan (http://www.targetscan.org/) and miRanda (microrna.org and miRbase). Finally, we found that the 3′-UTR of FOXA1 mRNA contained the complementary sequence of miR-212 (Figure 4A). This finding suggests that FOXA1, an important regulator of cell proliferation and apoptosis, may be a direct downstream target of miR-212. To confirm this prediction, we first examined the relationship between miR-212 and FOXA1 expression in HCC cases. As presented in Figure 4B, the expression of FOXA1 in miR-212 high-expressing HCC tissues was significantly lower than that in miR-212 low-expressing cases (*P* < 0.01). Next, Western blot analysis was performed to determine the effect of altering miR-212 expression on FOXA1 abundance in HCC cells. Additionally, we examined the expression of alpha-fetoprotein (AFP) and Yes-associated protein (YAP), of which the expression could be regulated by FOXA1. Overexpression of miR-212 in HepG2 cells dramatically reduced the expression of FOXA1 (*P* < 0.01, Figure 4C). The expression of AFP and YAP, the downstream targets of FOXA1, were reduced accordingly in miR-212 overexpressing HepG2 cells (*P* < 0.01, respectively, Figure 4C). In contrast, down-regulation of miR-212 resulted in increased expression of FOXA1, AFP and YAP in Bel-7402 cells (*P* < 0.01, respectively, Figure 4C). Furthermore, we confirmed the regulatory effect of miR-212 on FOXA1, AFP and YAP in xenograft tumor tissue (Supplementary Figure 1). These results confirmed that miR-212 inversely regulated FOXA1 abundance in HCC cells. Herein, we performed dual-luciferase reporter gene assays to determine whether miR-212 could directly target 3′-UTR of FOXA1 mRNA to regulate its expression. As expected, miR-212 significantly inhibited the luciferase activity of FOXA1 containing a wild-type (wt) 3′-UTR but did not suppress activity of FOXA1 with a mutant (mt) 3′-UTR (*P* < 0.01, Figure 4D). When anti-miR-212 was transfected, a significant increase in luciferase activity of wt FOXA1 3′-UTR was observed. However, transfection of anti-miR-212 did not lead to obvious alteration of the luciferase activity of FOXA1 containing mt FOXA1 3′-UTR (*P* < 0.01, Figure 4D). These data strongly suggest FOXA1 is a direct downstream target of miR-212 in HCC.

### Altering expression of FOXA1 influences the effect of miR-212 on HCC Cells

**Figure 5:**
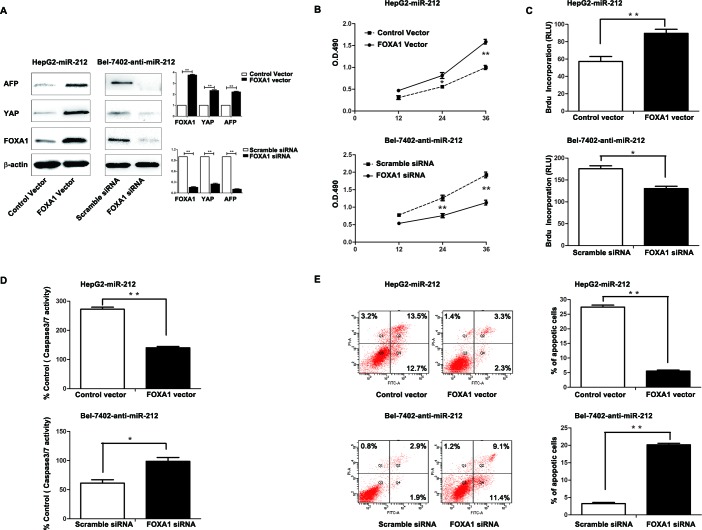
Altering FOXA1 expression partly abrogated the effect of miR-212 on HCC cells **A.** The FOXA1 expression plasmid significantly upregulated the expression of FOXA1 in miR-212 overexpressing HepG2 cells (HepG2-miR-212), while FOXA1 specific siRNA effectively downregulated FOXA1 expression in Bel-7402 cells transfected with miR-212 inhibitors (Bel-7402-anti-miR-212). *n* = three repeats with similar results, ** *P* < 0.01 by *t* test. **B-E.** Alteration of FOXA1 expression partly abolished the functional effect of miR-212 on cell viability, proliferation, capasase3/7 activity and the percentage of apoptotic cells of HepG2 and Bel-7402 cells. n = three repeats with similar results, *P < 0.05, ** *P* < 0.01 by *t* test (BrdU incorporation, caspase-3/7 activity and flow cytometry assays) and ANOVA (MTT assay).

To further determine whether FOXA1 is a functional target of miR-212, we restored the FOXA1 expression in HepG2-miR-212 cells by transfecting FOXA1 expression plasmid. FOXA1 expression was significantly up-regulated in HepG2-miR-212 cells after FOXA1 plasmid transfection, and resulted in obvious up-regulation of YAP and AFP (*P* < 0.01, respectively, Figure 5A). Functionally, restoration of FOXA1 expression in HepG2-miR-212 cells partially abrogated the effect of exogenous miR-212, resulting in significant increase of cell viability and proliferation (*P* < 0.01, respectively, Figure 5B and 5C) and obvious decrease of apoptosis (*P* < 0.01, respectively, Figure 5D and 5E). As a control group, HepG2-miR-control cells transfected with FOXA1 expression plasmid showed similar change of protein expression and cell function (Supplementary Figure 2). Similarly, silencing of FOXA1 in Bel-7402-anti-miR-212 cells partially abolished the effect of anit-miR-212 on cell viability, proliferation and apoptosis (*P* < 0.05, respectively, Figure 5B - 5E). These results demonstrate that FOXA1 is a downstream mediator for the function of miR-212 in HCC.

### Clinical significance of miR-212 and FOXA1 in HCC patients

**Figure 6:**
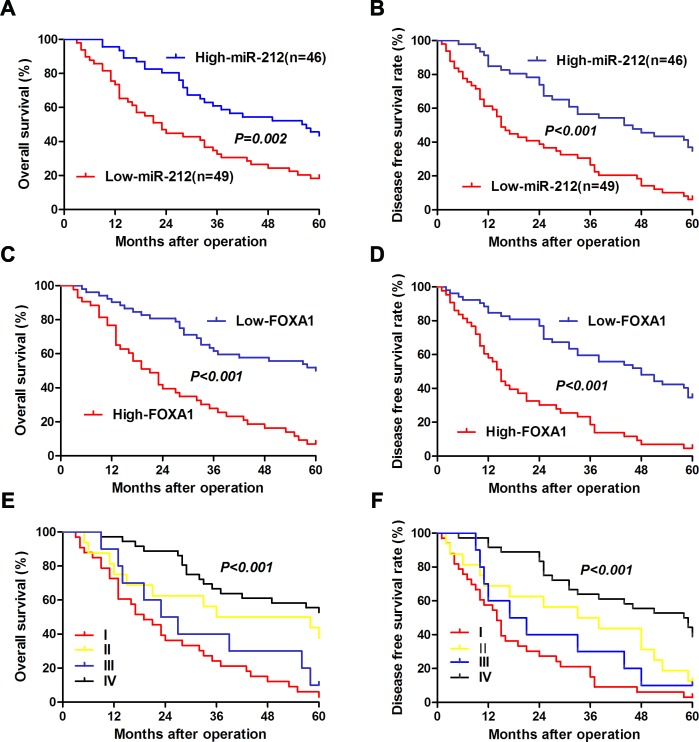
The prognostic value of miR-212 and FOXA1 for HCC patients assessed by Kaplen-Merier analysis HCC patients with high expression of miR-212 had better **A.** overall survival (OS) and **B.** disease free survival (DFS). HCC Patients with high expression of FOXA1 had shorter **C.** OS and **D.** DFS. HCC Patients in subgroup I had shortest **E.** OS and **F.** DFS, which were divided based on the combination of miR-212 and FOXA1 expression (subgroup I: low miR-212/high FOXA1; subgroup II: low miR-212/low FOXA1; subgroup III: high miR-212/high FOXA1; subgroup IV: high miR-212/low FOXA1). For each cohort, subgroups were divided according to the cutoff values of miR-212 and FOXA1, which was defined as the mean value of the cohort.

After confirming the functional role of miR-212 and FOXA1 in HCC, we further evaluated their clinical significance in 95 HCC patients. As shown in Table 1, decreased level of miR-212 was associated with high serum AFP level (*P* = 0.032), large tumor size (*P* = 0.018) and advanced TNM tumor stage (*P* = 0.031). Meanwhile, increased expression of FOXA1 was correlated with large tumor size (*P* < 0.001), venous infiltration (*P* = 0.002), high Edmondson-Steiner grading (*P* = 0.028), and advanced TNM stage (*P* < 0.001). These results indicate that aberrant expression of miR-212 and FOXA1 is correlated with poor clinical features of HCC patients. Furthermore, we investigated the prognostic value of miR-212 and FOXA1. Compared with patients with high level of miR-212, patients with low expression of miR-212 had shorter overall survival (OS) (*P* = 0.002, Figure 6A) and disease free survival (DFS) (*P* < 0.001, Figure 6B). Otherwise, OS and DFS of patients in the high FOXA1 group were significantly decreased (*P* < 0.001, respectively, Figure 6C and 6D). Multivariate Cox regression analysis showed that miR-212 level, FOXA1 expression, tumor size, TNM stage and Edmondenson stage were independent prognostic predictors for both OS and DFS (Table 2). Next, we divided patients into four subgroups based on miR-212 and FOXA1 expression levels. Patients with low expression of miR-212 and high expression of FOXA1 had the lowest OS and DFS (Figure 6E and 6F). In contrast, HCC patients with high expression of miR-212 and low expression of FOXA1 had the best OS and DFS (Figure 6E and 6F). Thus, combination of miR-212 and FOXA1 was an independent prognostic predictor for OS and DFS in HCC.

**Table 1:** Correlation between the clinicopathologic characteristics and miR-212 and FOXA1 expression in HCC

Characteristics	Total No. of patients n=95	No. of patients		*P*	No. of patients		*P*
		miR-212^low^	miR-212^high^		FOXA1^low^	FOXA1^high^	
Age (y)	<50	29	17	12	0.363	15	14	1.000
	≥50	66	32	34		37	29	
Sex	Male	70	40	30	0.069	34	36	0.061
	Female	25	9	16		18	7	
HBV	Absent	31	19	12	0.187	17	14	1.000
	Present	64	30	34		35	29	
Serum AFP level (ng/mL)	<400	37	14	23	0.032*	25	12	0.058
	≥400	58	35	23		27	31	
Tumor size (cm)	<5	36	13	23	0.018*	31	15	<0.001*
	≥5	59	36	23		21	28	
No. of tumor nodules	1	58	33	25	0.194	33	25	0.673
	≥2	37	16	21		19	18	
Cirrhosis	Absent	40	22	18	0.569	21	19	0.835
	Present	55	27	28		31	24	
Venous infiltration	Absent	46	20	26	0.126	33	13	0.002*
	Present	49	29	20		19	30	
Edmondson-Steiner grading	I+II	32	14	18	0.504	23	9	0.028*
	III+IV	63	35	28		29	34	
TNM tumor stage	I+II	62	27	35	0.031*	42	20	<0.001*
	III+IV	33	22	11		10	23	

HCC, hepatocellular carcinoma; HBV, hepatitis B virus; AFP, alpha-fetoprotein; TNM, tumor-node-metastasis.

* Statistically significant.

**Table 2:** Multivariate analyses of factors associated with OS and DFS of HCC patients (n=95)

	Overall Survival			Disease free Survival		
Clinical Variables	HR	95% CI	P	HR	95% CI	P value
Multivariate Analyses*						
miR-212	1.665	1.007-2.754	**0.047**	1.648	1.008-2.695	**0.046**
FOXA1	0.489	0.288-0.830	**0.008**	0.490	0.297-0.809	**0.005**
TNM tumor stage	0.211	0.117-0.381	**<0.001**	0.248	0.141-0.434	**<0.001**
Edmondson grading	0.475	0.293-0.769	**0.007**	0.510	0.322-0.835	**0.002**
Tumor size	0.530	0.338-0.832	**0.006**	0.517	0.332-0.807	**0.004**
Multivariate Analyses**						
I verus IV	0.349	0.176-0.691	**0.003**	0.334	0.173-0.647	**0.001**
TNM tumor stage	0.232	0.111-0.487	**<0.001**	0.229	0.111-0.473	**<0.001**
Edmondson grading	0.411	0.239-0.707	**0.001**	0.410	0.237-0.708	**0.001**
Tumor size	0.482	0.285-0.815	**0.006**	0.510	0.303-0.858	**0.011**

OS: overall survival; DFS: disease free survival

I: subgroup of low miR-212 and high FOXA1

IV: subgroup of high miR-212 and low FOXA1

* Multivariate analyses of miR-212, FOXA1, TNM tumor stage, Edmondson grading, and Tumor size

** Multivariate analyses of the combination of miR-212 and FOXA1 expression, TNM tumor stage, Edmondson grading, and Tumor size

## DISCUSSION

Numerous studies have demonstrated the critical roles for miRNAs whereby they participate in the initiation and progression of human cancers. Exploration of cancer-specific miRNAs and their downstream targets contributes to the identification of novel biomarkers and therapeutic targets for human cancers. In this study, we initially evaluated the expression of miR-212 in 40 paired samples of HCC and non-tumor tissues. Our data showed that the expression of miR-212 was impaired in HCC tissues. Furthermore, miR-212 was reduced in HCC cell lines as compared with a normal hepatic cell line. These results indicate that miR-212 may be a novel tumor suppressor and may play a critical role in hepatocarcinogenesis. Sustaining proliferation and resistance of cell death are two prominent hallmarks of cancer cells [25]. MiR-212 has been found to inhibit proliferation of gastric cancer by repressing retinoblastoma binding protein-2 [13]. And in NSCLC, it negatively regulates the anti-apoptotic protein PED and increases TRAIL-induced cell death [12]. But another study reported that miR-212 promoted pancreatic cancer cell proliferation by inhibiting patched-1 in pancreatic cancer [16]. Therefore, the influence of miR-212 on proliferation and apoptosis of malignant cells seems to be elusive and may be dependent on the type of cancer cells. In this study, with *in vitro* and *in vivo* experiments, we confirmed that miR-212 inhibited cell viability and proliferation, and promoted apoptosis of HCC cells.

To further understand the underlying mechanisms by which miR-212 exerts its biological effects on HCC cells, it is necessary to identify its downstream functional targets. FOXA1, an important regulator of cell proliferation and apoptosis, has been found to promote the development of HCC in male mice [23]. Elevated expression of FOXA1 has been observed in liver cancer cells [26]. Moreover, two public databases (TargetScan and Miranda) facilitated us to find out that FOXA1 was one of the predicted targets of miR-212. In this study, we confirmed that FOXA1 was a direct downstream target of miR-212 and it was implicated in the functional effect of miR-212 on HCC. An inverse correlation between the expression of miR-212 and FOXA1 protein was observed in HCC tissues. Up-regulation of miR-212 significantly reduced the expression of FOXA1 in HepG2 cells, whereas down-regulation of miR-212 increased the expression of FOXA1 in Bel-7402 cells. Overexpression of miR-212 decreased the luciferase reporter activity of FOXA1 wt 3′-UTR but not mt 3′-UTR. Finally, we confirmed that restoration of FOXA1 expression partly abrogated the functional effect of miR-212 on HCC cell viability, proliferation and apoptosis. Taken together, these data provide solid evidences to support that miR-212 exerts it inhibitory effect on HCC, at least in part, through inhibiting FOXA1.

Furthermore, it is interesting to mention that AFP is a well-defined downstream target of FOXA1 [27], and a widely used biomarker for the early detection and diagnosis of HCC [28]. Our study indicated that miR-212 decreased the expression of AFP by inhibiting FOXA1. Due to its association with AFP, miR-212 can potentially serve as a diagnostic biomarker for HCC. Therefore, it is worth to examine the sensitivity, specificity, and ROC on miR-212 as a diagnostic biomarker for HCC in further study. A recent study showed that YAP was a novel downstream target of FOXA1 [24]. And YAP overexpression is an early event in liver tumorigenesis [29] and can serve as an independent prognostic indicator for HCC patients [30]. Our data confirmed that miR-212 down-regulated YAP expression by inhibiting FOXA1. Therefore, miR-212/FOXA1/YAP pathway potentially plays an important role in HCC. Further experiments are needed to determine the function of this pathway in HCC.

The critical role of miR-212 and FOXA1 in the tumor growth and their aberrant expressions in HCC tissues promoted us to examine their clinical significance. Interestingly, we confirmed, for the first time, that decreased level of miR-212 and elevated expression of FOXA1 were associated with poor clinical features of HCC. Furthermore, we confirmed that miR-212, FOXA1 and the combination of miR-212 and FOXA1 were independent prognostic indicators for OS and DFS of HCC patients. And the prognostic significance of the combination of miR-212 and FOXA1 was more valuable than miR-212 or FOXA1 alone.

Currently, restoration of tumor suppressive miRNAs expression seems to be a promising strategy for cancer treatment [3]. Using the mimics for miR-34a and let-7 [31] or lentiviral vectors encoding let-7 [32] has achieved remarkable therapeutic benefit in both murine and human NSCLC. Thus, miR-212 mimics or viral vectors encoding miR-212 may also become the treatment options for HCC in the future. Moreover, results of our *in vitro* and *in vivo* experiments suggest that miR-212 may be a therapeutic target for HCC. Therefore, comparing the advantage of miR-212 targeted therapies to conventional and targeted drugs (e.g. sorafenib) for HCC will be of great importance in further study.

In conclusion, we find that miR-212 is down-regulated in HCC and its decreased expression is associated with poor prognostic features. *In vitro* and *in vivo* studies indicate that miR-212 inhibits tumor growth by inhibiting HCC cell proliferation and promoting apoptosis. Mechanistically, we suggest that miR-212 inhibits HCC cell viability and proliferation, and induces apoptosis by suppressing FOXA1. Notably, miR-212, FOXA1, and the combination of miR-212 and FOXA1 are independent prognostic factors for OS and DFS of HCC patients. Taken together, we consider that miR-212 may potentially act as a clinical biomarker, and may also be a therapeutic target, in HCC.

## MATERIALS AND METHODS

### Clinical samples and data

A total of 95 HCC samples and paired normal tumor-adjacent samples (>2cm distance from the margin of resection) were collected and used after obtaining informed consent. All patients received curative resection of their primary HCC in the Department of Hepatobiliary Surgery at the First Affiliated Hospital of Xi'an Jiaotong University during January 2006 to December 2008 with a median follow-up time of 38.7 months. All enrolled patients did not receive any perioperative chemotherapy or embolization. All protocols of this study are approved by the Ethics Committee of Xi'an Jiaotong University.

Patients' demographic and clinicopathologic data was obtained through review of hospital records. And disease recurrence and survival information was updated at each follow-up visit. The time between surgery date and first disease recurrence date was calculated as DFS. The time between the diagnostic biopsy or surgery date to death or last follow-up was determined as OS duration. The demographic features and clinicopathologic characteristics are presented in Table 1. Then, we divided these enrolled patients into different subgroups based on the expression of miR-212 and FOXA1. The clinical features and survival information were compared between groups to determine the clinical significance and prognostic value of miR-212 and FOXA1.

### Cell lines and transfection

Four HCC cell lines (HepG2, Huh7, Hep3B, and Bel-7402), and the human immortalized normal hepatocyte cell line (LO2) were obtained from the Institute of Biochemistry and Cell Biology, Chinese Academy of Sciences, Shanghai, China. All cells were cultured in complete Dulbecco's modified Eagle medium (DMEM, Gibco, Grand Island, NY, USA) containing 10% fetal bovine serum (FBS, Gibco) with 100 units/mL penicillin and 100 μg/mL streptomycin (Sigma, St-Louis, MO, USA) in a humidified containing of 5% CO2 incubator at 37°C.

MiRNA vectors, including miR-212 expression vector (HmiR0269-MR04), the control vector for miR-212 (CmiR0001-MR04), miR-212 inhibitor (HmiR-AN0319-AM04) and the negative control for the miR-212 inhibitor (CmiR-AN0001-AM04), were purchased from Genecopoeia (Guangzhou, China). Plasmids carrying human FOXA1 were purchased from OriGene (SC108256, Beijing, China). Cells were seeded at 1 × 10^5^ cells per well in a six-well plate and transfected with synthetic miRNA vectors and FOXA1-expression plasmid using Lipofectamine 2000 according to the manufacturer's instructions (Invitrogen, Carlsbad, CA, USA).

### Immunohistochemical staining

Immunohistochemistry was performed on paraformaldehyde-fixed paraffin sections. Paraffin embedded samples were cut into 4 μm-thick sections, which were baked at 60 °C for at least 6 hours. Paraffin sections were then deparaffinized in xylene and re-hydrated through graded ethanol. Antigen retrieval was performed in sodium citrate buffer for 2 minutes in a pressure cooker, and then these slices were quenched for endogenous peroxidase activity in 3% hydrogen peroxide for 10 min. They were blocked with goat plasma at 37 °C for 30 minutes and incubated with FOXA1 antibody (1:100, #5089, Abcam, Cambridge, MA) or Ki-67 antibody (1:100, #9027, Cell Signaling, Danvers, MA, USA) at 4 °C overnight. The biotinylated secondary antibody (ZSGB-Bio, Beijing, China) was used to detect the primary antibody. Then sections were incubated with diaminobenzidine before being counterstained with hematoxylin. At last, they were dehydrated in graded ethanol and transparentized in xylene. The percentage of positive tumor cells or hepatocytes was graded as per the following criteria: 0, less than10%; 1, 10–30%; 2, 31–50%; 3, more than 50%.

### Immunoblotting

Cells and xenograft tumor tissues were lysed in RIPA buffer (50 mM Tris pH 7.5, 150 mM NaCl, 1% TritonX-100, 5 mM ethylenediaminetetraacetic acid). Protein concentration was determined using the BCA Kit (Pierce, IL, USA). Protein samples (30 ug) were separated by sodium dodecyl sulfate-polyacrylamide gel electrophoresis and transferred onto a nitrocellulose membrane. The blots were then probed with antibodies against the following primary antibodies: FOXA1 (1:1000), YAP (1:1000, #12395, Cell signaling, Danvers, MA, USA), AFP (1:1500, #3903, Cell signaling, Danvers, MA, USA) and β-actin (1:1000, #12262, Cell signaling, Danvers, MA, USA). Blots were incubated with horseradish peroxidase-conjugated goat anti-mouse or anti-rabbit secondary antibodies (1:5000-1:10000, Bio-Rad, Hercules, CA, USA) and detected using the Bio-Rad Gel imaging system.

### Real-time quantitative reverse transcription polymerase chain reaction (qRT-PCR)

The PCR amplification for the quantification of the miR-212 and U6 was performed using TaqMan miRNA Reverse Transcription Kit (Applied Biosystms, Foster City, CA, USA) and TaqMan Human MiRNA Assay Kit (Applied Biosystems). The relative expression of miR-212 was shown as fold difference relative to U6.

### Cell viability and proliferation assays

2 × 10^3^ HCC cells were seed in 96-well plates and the 3-(4, 5-dimethylthiazol-2-yl)-2, 5-diphenyl tetrazolium bromide (MTT, Roche, USA) assay was employed to assess cell viability at 12, 24 and 36 hours. For proliferation assay, a Cell Proliferation ELISA, BrdU (5-bromodeoxyuridine) (chemiluminescent) (Roche, USA) was used as previously described [33].

### Cell apoptosis detection

The apoptosis of cells was evaluated by the Annexin-V-FLUOS Staining Kit (Roche, USA), as previously described [34]. The caspase-3/7 activity assay was measured using an Apo-ONE® Homogeneous Caspase-3/7 Assay (Promega, Madison, WI, USA), as described in our previous study [35].

### Luciferase reporter assay

The 3′-UTR sequence of FOXA1 predicted to interact with miR-212 or the mutated sequence within the predicted target sites was synthesized and inserted into the pGL3 control vector (Promega, Madison, WI, USA). These constructs were named as wt FOXA1-3′UTR or mt FOXA1-3′UTR, respectively. Then, HepG2 cells (1 × 10^5^) were seeded into 24-well plates, and were cultured in OptimMEM reduced serum media (Life Technologies) as per the recommended conditions, and were cotransfected with 200 ng of each luciferase reporter construct (the wt or mt 3′-UTR of FOXA1 mRNA) and miR-212 expression vector, miR-212 inhibitor, control vector or negative control (50 nM) using Fugene (Promega, Madison, WI, USA). After 48 h, the cells were harvested and luciferase activity was measured using the dual-luciferase reporter assay system (Promega, Madison, WI, USA). Firefly luciferase activity was normalized to the Renilla luciferase activity. Results were obtained from three independent experiments performed in triplicate.

### *In vivo* experiments

4-6 week-old female BALB/c nude mice (Centre of Laboratory Animals, The Medical College of Xi'an Jiaotong University, Xi'an, China) were used to establish the nude mouse xenograft model. 5 × 10^6^ HepG2 cells transfected with miR-212 expressing or control vectors were mixed in 150 uL of Matrigel and were inoculated subcutaneously into the flank of nude mouse. Tumor volume was determined by measuring two of its dimensions with calipers every 7 days, and then calculated as tumor volume = length × width × width/2. All mice were sacrificed at 3 weeks after the injection of HCC cells. The xenograft tumor tissues were explanted for pathological examination. Apoptosis cells in the isolated tumor tissues were detected using the TUNEL assay as described before. All *in vivo* protocols were approved by the Institutional Animal Care and Use Committee of Xi'an Jiaotong University.

### Statistical analysis

Results are presented as mean ± S.E.M. The SPSS statistical package for Windows Version 13 (SPSS, Chicago, IL, USA) and GraphPad Prism 5 software (GraphPad Software, Inc, San Diego, CA, USA) were used for the Pearson chi-squared test, a two-tailed Student's *t* test, a Kaplan–Meier plot, a log-rank test or an ANOVA when appropriate. *P* < 0.05 was considered to be statistically significant.

## SUPPLEMENTARY MATERIAL FIGURES


