# Supplementary material for: MicroRNA-212 suppresses tumor growth of human hepatocellular carcinoma by targeting FOXA1
Source: Oncotarget. 2015 Apr 23;6(15):13216–28. doi: 10.18632/oncotarget.3916 (PMC4537009; doi:10.18632/oncotarget.3916)
Supplement: Supplementary file 1 [file oncotarget-06-13216-s001.pdf]

MicroRNA-212 suppresses tumor growth of human hepatocellular carcinoma by targeting FOXA1

Supplementary Material

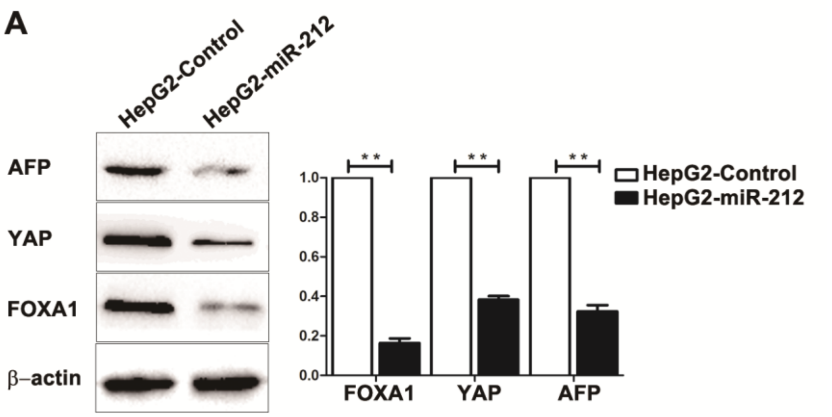

Supplementary figure 1: The expression level of FOXA1, YAP and AFP in xenograft tumor tissues. (A) HepG2 cells that were transfected with miR-212 expression vector (miR-212 group) or control vector (miR-control group) were implanted into nude mice via subcutaneous injection. Xenografe tumor tissues isolated from miR-212 or miR-control group were subjected to Western blot for FOXA1, YAP and AFP expression. The expressions of FOXA1, YAP and AFP in miR-212 group were significantly lower than those in miR-control group. n = six, ** $P < 0.01$ by *t* test.

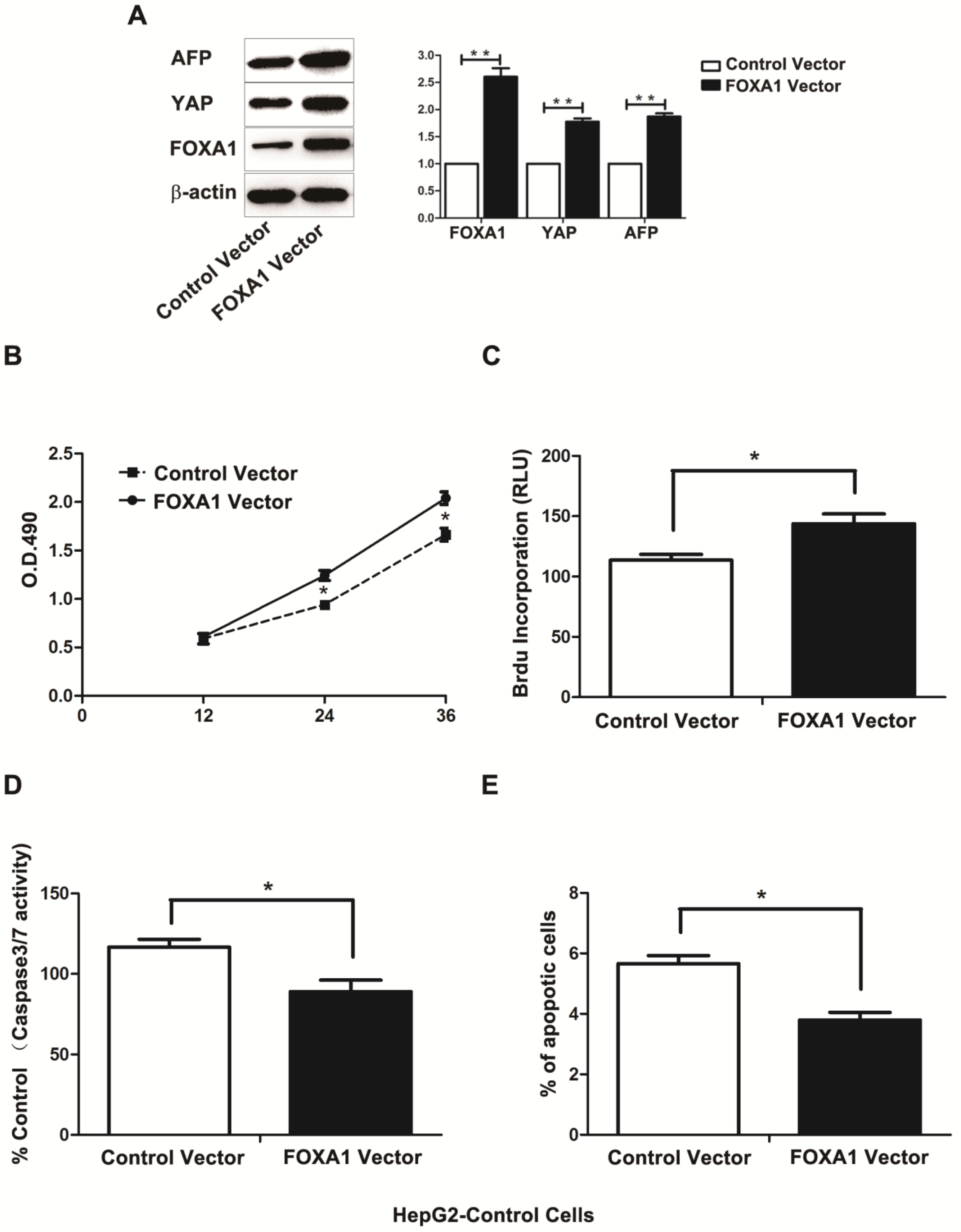
A
AFP
YAP
FOXA1
β-actin
Control Vector
FOXA1 Vector
3.0
2.5
2.0
1.5
1.0
0.5
0.0
**
FOXA1
YAP
AFP
Control Vector
FOXA1 Vector
B
Control Vector
FOXA1 Vector
O.D.490
0
12
24
36
*
C
200
150
100
50
0
Brdu Incorporation (RLU)
*
Control Vector
FOXA1 Vector
D
% Control （Caspase3/7 activity)
150
100
50
0
*
Control Vector
FOXA1 Vector
E
% of apoptotic cells
8
6
4
2
0
*
Control Vector
FOXA1 Vector
HepG2-Control Cells

Supplementary figure 2: The effects of FOXA1 overexpression on miR-control transfected HepG2 cells. (A) HepG2 cells that were transduced with miR-control vector (miR-control) were transfected with FOXA1 or control vector. FOXA1 overexpression increased the levels of YAP and AFP protein, as determined by Western blot. * $P < 0.05$ by *t* test. (B-E) Overexpression of FOXA1 significantly affected the cell viability, proliferation, caspase-3/7 activity, and the percentage of apoptotic cells in miR-control transfected HepG2 cells. n = three repeats with similar results, * $P < 0.05$ by *t* test (BrdU incorporation, caspase-3/7 activity and flow cytometry assays) and ANOVA (MTT assay).
